# The X‐linked juvenile retinoschisis protein retinoschisin is a novel regulator of mitogen‐activated protein kinase signalling and apoptosis in the retina

**DOI:** 10.1111/jcmm.13019

**Published:** 2016-12-20

**Authors:** Karolina Plössl, Bernhard H.F. Weber, Ulrike Friedrich

**Affiliations:** ^1^ Institute of Human GeneticsUniversity of RegensburgRegensburgGermany

**Keywords:** X‐linked juvenile retinoschisis, retinoschisin, RS1, Na/K‐ATPase, MAP kinase signalling, apoptosis

## Abstract

X‐linked juvenile retinoschisis (XLRS) is a hereditary retinal dystrophy in young males, caused by mutations in the *RS1* gene. The function of the encoded protein, termed retinoschisin, and the molecular mechanisms underlying XLRS pathogenesis are still unresolved, although a direct interaction partner of the secreted retinoschisin, the retinal Na/K‐ATPase, was recently identified. Earlier gene expression studies in retinoschisin‐deficient (*Rs1h* ^*−/Y*^) mice provided a first indication of pathological up‐regulation of mitogen‐activated protein (MAP) kinase signalling in disease pathogenesis. To further investigate the role for retinoschisin in MAP kinase regulation, we exposed Y‐79 cells and murine *Rs1h* ^*−/Y*^ retinae to recombinant retinoschisin and the XLRS‐associated mutant RS1‐C59S. Although normal retinoschisin stably bound to retinal cells, RS1‐C59S exhibited a strongly reduced binding affinity. Simultaneously, exposure to normal retinoschisin significantly reduced phosphorylation of C‐RAF and MAP kinases ERK1/2 in Y‐79 cells and murine *Rs1h* ^*−/Y*^ retinae. Expression of MAP kinase target genes *C‐FOS* and *EGR1* was also down‐regulated in both model systems. Finally, retinoschisin treatment decreased pro‐apoptotic *BAX‐2* transcript levels in Y‐79 cells and *Rs1h* ^*−/Y*^ retinae. Upon retinoschisin treatment, these cells showed increased resistance against apoptosis, reflected by decreased caspase‐3 activity (in Y‐79 cells) and increased photoreceptor survival (in *Rs1h* ^*−/Y*^ retinal explants). RS1‐C59S did not influence C‐RAF or ERK1/2 activation, *C‐FOS* or *EGR1* expression, or apoptosis. Our data imply that retinoschisin is a novel regulator of MAP kinase signalling and exerts an anti‐apoptotic effect on retinal cells. We therefore discuss that disturbances of MAP kinase signalling by retinoschisin deficiency could be an initial step in XLRS pathogenesis.

## Introduction

Pathogenic alterations affecting the *RS1* gene on chromosome Xp22.1 have been shown to cause XLRS (OMIM #312700) 1, a macular degeneration disorder in young males with a prevalence of approximately 1:5000 to 1:20,000 2. Disorganization of retinal layers and distinct abnormalities in the electroretinogram (ERG) are hallmarks of the disease. Specifically, a characteristic splitting of retinal layers, presenting as a bilateral foveal schisis, is found at an early stage of the disease and results in cystic degeneration of the central retina 3, 4, 5, 6. Additionally, defects in signal transmission from photoreceptor to bipolar cells as visualized by ERG recordings are observed and reveal a characteristic reduction in the b‐wave amplitude, whereas the a‐wave remains almost unaffected 4, 7. Comparable pathological features are also evident in XLRS mice, generated *via* a targeted disruption of the murine orthologue of *RS1*, the *Rs1h* gene 8, 9, 10. Due to the close resemblance of the retinal phenotype in *Rs1h* knockout mice and XLRS patients, the retinoschisin‐deficient mouse represents an excellent disease model widely used in experimental studies addressing the mechanisms of XLRS pathology but also novel treatment approaches 11, 12, 13, 14, 15, 16.

The *RS1* gene is organized into six exons and encodes a 224‐amino acid (aa) precursor protein 1. It is specifically expressed in the retina by photoreceptor and bipolar cells, as well as in pinealocytes of the pineal gland 1, 17, 18. During protein synthesis, a 23‐aa signal sequence is cleaved to produce a 201‐aa mature polypeptide which is secreted from photoreceptors and bipolar cells as a homooctamer held together by intermolecular disulphide bonds between aa 223 and aa 59 19, 20, 21, 22. So far, over 190 unique XLRS‐associated sequence variants in *RS1* have been reported (Leiden Open Variation Database, http://grenada.lumc.nl/LOVD2/eye/home.php?select_db=RS1, accessed May 2016). Functional assessment of a subset of these variants demonstrated that the vast majority of mutations result in a complete loss of the functional protein 4.

Despite intensive research, the precise molecular function of retinoschisin remains unresolved. Searching for retinoschisin interaction partners, Molday *et al*. 14 identified the retina‐specific Na/K‐ATPase composed of the two subunits ATP1A3 (α3) and ATP1B2 (β2). Subsequently, our group confirmed the Na/K‐ATPase to be required for anchoring retinoschisin to plasma membranes 23. The Na/K‐ATPase is a plasma membrane spanning ion pump, responsible for maintaining the cellular membrane potential by transporting Na^+^ and K^+^ ions across the plasma membrane against their electrochemical gradient 24, 25. Despite this essential task, Na/K‐ATPases also mediate intercellular adhesion 26, 27, 28 and induce activation of intracellular signalling pathways upon binding of glycoside hormones such as ouabain 25, 29, 30, 31, 32, 33, 34. Members of the FXYD family, a class of Na/K‐ATPase‐binding proteins 35, 36, were reported to be important regulators of the Na/K‐ATPase, modulating its pump activity and mediation of intercellular adhesion 37, 38, 39. Similar to FXYD proteins, one could consider retinoschisin to exert a role as a modulator of Na/K‐ATPase activity.

A genomewide expression analysis of the *Rs1h*‐deficient (*Rs1h* ^*−/Y*^) murine retina first indicated an increased activation of the ERK pathway in early XLRS pathogenesis, prior to apoptotic photoreceptor degeneration 40. The ERK pathway is one of the four major MAP kinase pathways 41, 42 known to play a crucial role in fundamental developmental and physiological processes such as apoptosis, neuroprotection, neuronal development and adhesion 41, 43, 44, 45, 46, 47, 48, 49, 50. It is tempting to speculate that misregulation of MAP kinase signalling caused by retinoschisin deficiency could be an initial step in XLRS pathogenesis. However, aberrant MAP kinase activation could also be a secondary event, caused by alterations of the cellular/retinal homeostasis in the XLRS disease process.

In this study, we examined whether retinoschisin binding to retinal membranes directly modulates MAP kinase signalling. Our findings in cultured Y‐79 cells and in retinal explants of *Rs1h* ^*−/Y*^ mice demonstrate that the addition of recombinant retinoschisin, but not recombinant mutant retinoschisin, significantly down‐regulates MAP kinase signalling*,* as well as protects against apoptosis. We conclude that retinoschisin deficiency could be a trigger for disease pathogenesis by a defective control of MAP kinase signalling and apoptosis in the retina.

## Materials and methods

### Animal models

The *Rs1h* ^*−/Y*^ mouse was generated as described earlier 9 and kept on a C57BL/6 background. Mice were housed under specific pathogen‐free barrier conditions at the Central Animal Facility of the University of Regensburg and maintained under conditions established by the institution for their use, in strict compliance with NIH guidelines. Mice were sacrificed 10 or 18 days after birth by decapitation or cervical dislocation after inhalation of carbon dioxide, respectively.

### Cell culture

Y‐79 and Weri‐Rb1 (ATCC, Manassas, VA, USA) cells were cultivated in RPMI medium with 10% FCS as well as 100 U/ml penicillin/streptomycin. ARPE‐19 cells (ATCC) were maintained in DMEM/Ham's F12 medium containing 10% FCS and 100 U/ml penicillin/streptomycin. BV‐2 cells were grown in RPMI‐1640 with 5% FCS, 100 U/ml penicillin/streptomycin and 195 nM β‐mercaptoethanol. Hek293 cells (Invitrogen, Carlsbad, CA, USA) were maintained in DMEM high glucose medium containing 10% FCS, 100 U/ml penicillin/streptomycin and 500 μg/ml G418. All media and cell culture supplies were purchased from Life Technologies (Carlsbad, CA, USA). Cell lines were grown in a 37°C incubator with a 5% CO_2_ environment and subcultured when they reached 90% confluency for Hek293, BV‐2 and ARPE‐19 or a concentration of 4–5 × 10^5^ cells/ml for Y‐79 and Weri‐Rb1. Only Y‐79 cells passaged less than 10 times were applied in signalling or apoptosis assays.

### RNA analysis

RNA was isolated from cell lines using the Qiagen RNeasy Mini Kit (Qiagen, Venlo, the Netherlands). RNA from murine retinae and cultured retinal explants was isolated using the PureLink^™^ RNA Micro Kit (Invitrogen), according to the manufacturers’ protocols. One microgram of total RNA was transcribed into cDNA using RevertAid M‐MuLV Reverse Transcriptase (Fermentas, St Leon‐Rot, Germany) and poly(dT) primers according to the manufacturer's instructions. Semiquantitative RT‐PCR was performed as described by 23, with primers given in Table S1. Quantitative real‐time RT‐PCR was performed and analysed as published 51 with primers given in Table S1.

### Western blot analysis

Proteins were separated after application of Laemmli buffer 52 on 12.5% gels or gradient gels 4–20% Mini‐PROTEAN^®^ TGX^™^ Precast Protein Gels (Bio‐Rad Laboratories, Hercules, CA, USA; for analysis of retinoschisin octamers). For Western blotting, proteins were transferred to polyvinylidene difluoride (PVDF) membranes (Immobilon; Millipore, Schwalbach, Germany) Antibodies were used as follows: Antibodies against Myc tag, phospho‐c‐Raf (Ser338), c‐Raf and phospho‐44/42‐MAPK (Erk1/2; Thr202/Tyr204) were obtained from Cell Signaling Technologies (Danvers, MA, USA). Antibodies against ACTB and ERK1/2 were from Sigma‐Aldrich (St. Louis, MO, USA). Secondary anti‐rabbit or antimouse IgG horseradish peroxidase (HRP)‐linked antibodies were from Calbiochem (Merck Chemicals GmbH, Schwalbach, Germany). Antibody dilutions were applied according to the manufacturer's recommendations. RS1 primary antibody (diluted 1:10,000) was kindly provided by Prof. Robert Molday, University of British Columbia, Vancouver, Canada. Clarity^™^ Western ECL Substrate (Bio‐Rad Laboratories) and an Odyssey FC imager (LI‐COR, Lincoln, NE, USA) were used to visualize Western blots. Densitometric evaluation of Western blots was carried out using ImageJ (imagej.nih.gov).

### Immunolabelling of retinal cryosections

Retinal explants were washed in PBS (2.7 mM KCl, 140 mM NaCl, 10 mM phosphate, pH 7.4) once. Subsequently, they were submerged in 4% (w/v) paraformaldehyde and incubated for 1 hr at room temperature. Retinae were washed in PBS twice before they were put in 30% (w/v) sucrose overnight. Single retinae were then embedded in Richard‐Allan Scientific^™^ Neg‐50^™^ Frozen Section Medium (Thermo Fisher Scientific, Waltham, MA, USA) and fast frozen in liquid nitrogen. About 10 μm cryosections were cut. Immunolabelling with anti‐ATP1B2 and antiretinoschisin antibodies was performed as described by 23. Cone visualization was performed with Alexa 488‐conjugated peanut agglutinin (1:250, PNA; Invitrogen). Rhodopsin staining was performed with Rho‐1D4 antibody (1:1000), kindly provided by Prof. Robert Molday, University of British Columbia, Vancouver, Canada. The sections were counterstained with 4′,6‐diamidino‐2‐phenylindol (DAPI, 1:1000; Molecular Probes, Leiden, the Netherlands). Images were taken with custom‐made VisiScope CSU‐X1 Confocal System (Visitron Systems, Puchheim, Germany) equipped with high‐resolution sCMOS camera (PCO AG, Kehlheim, Germany).

### Expression cloning

The coding sequence of non‐mutant retinoschisin (NM_000330.3) was amplified from cDNA of retinal tissue using oligonucleotide primers containing a *EcoRI* restriction site at the 5′ end and a *XhoI* restriction site at the 3′ end of the *RS1* coding sequence (for primer sequences, see Table S1). The coding sequence of the XLRS‐associated RS1 mutant RS1‐C59S (NM_000330.3 (RS1):c.175T>A [p.Cys59Ser] http://grenada.lumc.nl/LOVD2/eye/home.php?select_db=RS1) was generated by site‐directed mutagenesis on the retinoschisin coding sequence (primer sequences shown in Table S1). For purification, the two RS1 variants were each tagged with an N‐terminal Myc tag, following the leader sequence (after aa 23). This peptide insertion into the full‐length *RS1* coding sequence was performed by fusing the N‐terminal part of both *RS1* coding sequences from positions 1 to 69 (aa 1–23) to the N‐terminal half of the Myc tag sequence (for primers, see Table S1). The C‐terminal part of both *RS1* coding sequences (aa 70 to stop codon) was fused to the C‐terminal half of the Myc tag sequence (for primers, see Table S1). C‐ and N‐terminal *RS1* fragments were ligated *via* a BclI restriction site which was introduced into the Myc tag, and inserted into the pCDNA3.1^™^ expression vector (Thermo Fisher Scientific).

### Expression and purification of recombinant *RS1* variants

Expression constructs were transfected into Hek293 cells using calcium phosphate transfection as described by 53. About 7 hrs after transfection, the culture medium was replaced by Opti‐MEM^®^ containing 100 U/ml penicillin/streptomycin (Life Technologies) and cells were cultured for additional 48 hrs.

Myc‐tagged retinoschisin and RS1‐C59S were isolated from cultivation media by immunoprecipitation using Pierce^™^ Anti‐c‐Myc Agarose (Thermo Fisher Scientific) according to the manufacturer's instructions. Concentrations of purified proteins were determined using the Bio‐Rad DC^™^ Protein Assay Kit (Bio‐Rad Laboratories).

For use as a treatment control, Hek293 cells were transfected with empty pCDNA3.1^™^ expression vector, and cultivation medium of these cells was subjected to purification procedure exactly like medium from cells transfected with RS1 variants.

Purity of purified Myc‐tagged RS1 proteins and control eluate was verified *via* silver staining, Coomassie Blue staining and Western blot analysis using antibodies against the Myc tag and against retinoschisin (Fig. S1).

### Binding of *RS1* protein variants to membranes

Retinoschisin binding to adherent cell lines (BV‐2, ARPE and Hek293) as well as to murine retinal membranes (P10) was assessed as described by 23, but with a prolonged incubation time of 1 hr.

Retinoschisin binding to suspension cell lines Y‐79 and Weri‐Rb1 was analysed by incubating 4 × 10^6^ cells in 5 ml RS1 containing medium (from supernatant of stably transfected Hek293 cells 23) for 1 hr, with subsequent washing steps as described 23.

For comparing binding affinity of retinoschisin and RS1‐C59S to Y‐79 cells and *Rs1h* ^*−/Y*^ murine retinal membranes, 6 μg of purified retinoschisin or RS1‐C59S was added to 5 ml cultivation medium and incubated for 10, 30 and 60 min. Subsequent steps were performed as above.

For localization of bound recombinant RS1 variants on *Rs1h* ^*−/Y*^ murine retinae, *Rs1h* ^*−/Y*^ murine retinal explants were incubated with 1 μg RS1, RS1‐C59S or control eluate, as described in signalling experiments. After 30 min. of incubation, the retinal tissue was washed with PBS once before immunolabelling of retinal cryosections was performed.

### Analysis of signalling pathways in retinal explants and Y‐79 cells

Y‐79 cells were grown to a concentration of 4–5 × 10^5^ cells/ml in 10 ml medium. The experiment was started by adding 1 μg purified retinoschisin or RS1‐C59S, or equal volume of control eluate. After 10 or 30 min. of incubation at 37°C, cells were harvested by centrifugation. For Western blotting, cells were resuspended in 200 μl of pre‐cooled PBS with PhosSTOP^™^ phosphatase inhibitor (Sigma‐Aldrich) and lysed by sonication (10 sec., 40% intensity). For RNA isolation, cells were washed once with pre‐cooled PBS before they were subjected to RNA isolation.

Eyes from *Rs1h* ^*−/Y*^ mice at post‐natal day 10 were enucleated and retinal explants were dissected as described by 54. Retinae were incubated in 800 μl DMEM/Ham's F12 containing 10% FCS, 100 U/ml penicillin/streptomycin, 2 mM l‐glutamine and 2 μg/ml insulin (Thermo Fisher Scientific). One microgram purified retinoschisin or RS1‐C59S or equal volumes of control eluate were added. After 10 or 30 min. of incubation at 37°C, retinal explants were removed from medium and transferred to 200 μl of pre‐cooled PBS containing PhosSTOP^™^ phosphatase inhibitor. For Western blot analysis, retinal explants were sonicated for 10 sec. at 40% intensity. For RNA isolation, retinal explants were immediately transferred into lysis buffer (PureLink^™^ RNA Micro Kit; Invitrogen).

### Analysis of caspase‐3 activity in Y‐79 cells

About 2 × 10^6^ cells/well were seeded onto poly‐l‐lysine‐coated 24‐well plates. Cells were allowed to adhere overnight before 0.1 μg of purified retinoschisin or RS1‐C59S, or equal volumes of control eluate were added to 1 ml medium per well. After 1 hr, the culture medium was changed to 1 ml RPMI (containing RS1 variants or control eluate as before) and 0.2 mM H_2_O_2_ to induce apoptosis or 0 mM H_2_O_2_ as control. After 2 hrs, the medium was replaced by 1 ml RPMI containing RS1 variants or control eluate as before. Cells were allowed to recover for 18 hrs before they were subjected to a caspase‐3 activity test using the EnzChek^®^ Caspase‐3 Assay Kit #2 by Thermo Fisher Scientific according to the manufacturer's directions.

### Analysis of photoreceptor degeneration in retinal explants

Eyes were enucleated from mice at post‐natal day 18 and retinae were dissected as described before 55. Five retinae each were subjected to treatment with retinoschisin, RS1‐C59S and control protein. Retinal explants were transferred into pre‐warmed medium (DMEM/Ham's F12 containing 10% FCS, 100 U/ml antibiotic–antimytotic, 2 mM l‐glutamine and 2 μg/ml insulin, all from Life Technologies) immediately after preparation, rinsed once in pre‐warmed medium and then transferred onto Track Etch Membrane Filters (Whatman plc, Maidstone, UK) in 35‐mm tissue culture dishes containing 3 ml of medium to which 1 μg of purified retinoschisin, RS1‐C59S or control eluate had been added. The retinal explants on the filters were covered in a small droplet of medium. Cultivation was carried out under sterile conditions in a 37°C incubator with a 5% CO_2_ environment. Medium was replaced every 36 hrs and total cultivation time was 1 week. Subsequently, the retinal explants were fixed, cryopreserved and cut into 10‐μm sections for subsequent histological analyses as described before. After PNA staining, cones were counted in each two different sections of the same retina, in 200‐nm‐wide regions to the left and the right side of the optic nerve. Rods were stained with anti‐Rho‐1D4 antibody. Staining signals of each two different sections of the same retina, in 200‐nm‐wide regions to the left and the right side of the optic nerve, were quantified using ImageJ.

## Results

### Increased MAP kinase signalling in murine *Rs1h* ^*−/Y*^ retinae

A study by Gehrig *et al*. 40 previously indicated up‐regulated MAP kinase activity in early retinal development of *Rs1h* ^*−/Y*^ mice. The authors found in the murine *Rs1h* ^*−/Y*^ retinae increased phosphorylation of extracellular‐signal‐regulated kinases 1 and 2 (Erk1/2), as well as up‐regulated expression of *Egr1* (early growth response protein 1), a prominent target gene of activated MAP kinases 56.

To first verify these results, we analysed phosphorylation of Erk1/2 as well as *Egr1* expression in retinae of wild‐type and *Rs1h* ^*−/Y*^ mice, 7, 10 and 14 days after birth (P7, P10 and P14). Furthermore, we investigated phosphorylation of c‐Raf, a central constituent of the ERK pathway, the activation of which precedes and is required for Erk1/2 phosphorylation 57, in P7, P10 and P14 retinae. As an additional MAP kinase target gene, we assessed expression of the FBJ murine osteosarcoma viral oncogene homologue gene (*c‐Fos)*, which is expressed in response to transient and sustained ERK signalling 56, 58, 59, 60, 61.

**Figure 1:**
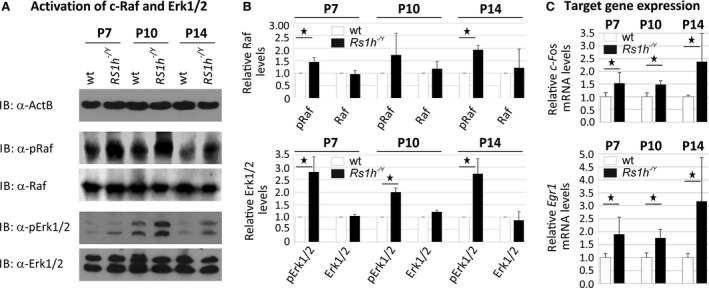
Influence of retinoschisin deficiency on MAP kinase signalling in the murine retina. C**‐**Raf and Erk1/2 phosphorylation in murine wild‐type and *Rs1h* ^*−/Y*^ retinae, harvested at post‐natal days 7, 10 and 14 (P7, P10 and P14). Retinal lysates were subjected to Western blot analyses with antibodies against phosphorylated c‐Raf (pRaf), total c‐Raf (Raf), phosphorylated Erk1 and Erk2 (pErk1/2), total Erk1 and Erk2 (Erk1/2), as well as ActB as a control (**A**). Densitometric quantification (**B**) was performed with immunoblots from three independent sample sets. Signals for pErk1/2, Erk1/2, pRaf and Raf were normalized against ActB and calibrated against signals for wild‐type retinae. Data represent the mean + S.D. (**C**) *C‐Fos* and *Egr1* expression in murine wild‐type and *Rs1h* ^*−/Y*^ retinae harvested at post‐natal days 7, 10 and 14 (P7, P10 and P14). mRNA expression of *C‐Fos* and *Egr1* was determined *via* quantitative real‐time RT‐PCR. Five independent sample sets were analysed. Results were normalized to *Hprt* transcript levels and calibrated with the control. The mean + S.D. for the three (immunoblot analyses) or five (quantitative RT‐PCR) independent sample sets is given. Asterisks mark statistically significant (*P < 0.05) and highly significant (**P < 0.01) differences.

Western blot analyses showed an increase in c‐Raf and Erk1/2 phosphorylation in *Rs1h* ^*−/Y*^ retinae compared with wild‐type retina (Fig. 1A). C‐Raf phosphorylation levels in *Rs1h* ^*−/Y*^ retinae increased to around 150–200%, whereas Erk1/2 phosphorylation levels rose to around 250–300% above normal (Fig. 1B). These differences were obtained in all stages (P7, P10 and P14), and each were statistically significant (*P* < 0.05, except for c‐Raf at P10; Fig. 1A and B) Retinoschisin deficiency had no influence on levels of total c‐Raf and Erk1/2, independent of the post‐natal stages.

Expression of MAP kinase target genes *C‐Fos* and *Egr1* was increased in *Rs1h* ^*−/Y*^ retinae, at all developmental stages; *c‐Fos* expression levels in *Rs1h* ^*−/Y*^ retinae were between 150% and 250%, and *Egr1* expression levels between 175% and 300% compared with wild‐type retinae. Differences in expression were statistically significant between wild‐type and *Rs1h* ^*−/Y*^ retinae (*P* < 0.05).

### Binding of retinoschisin to different retinal cell types

**Figure 2:**
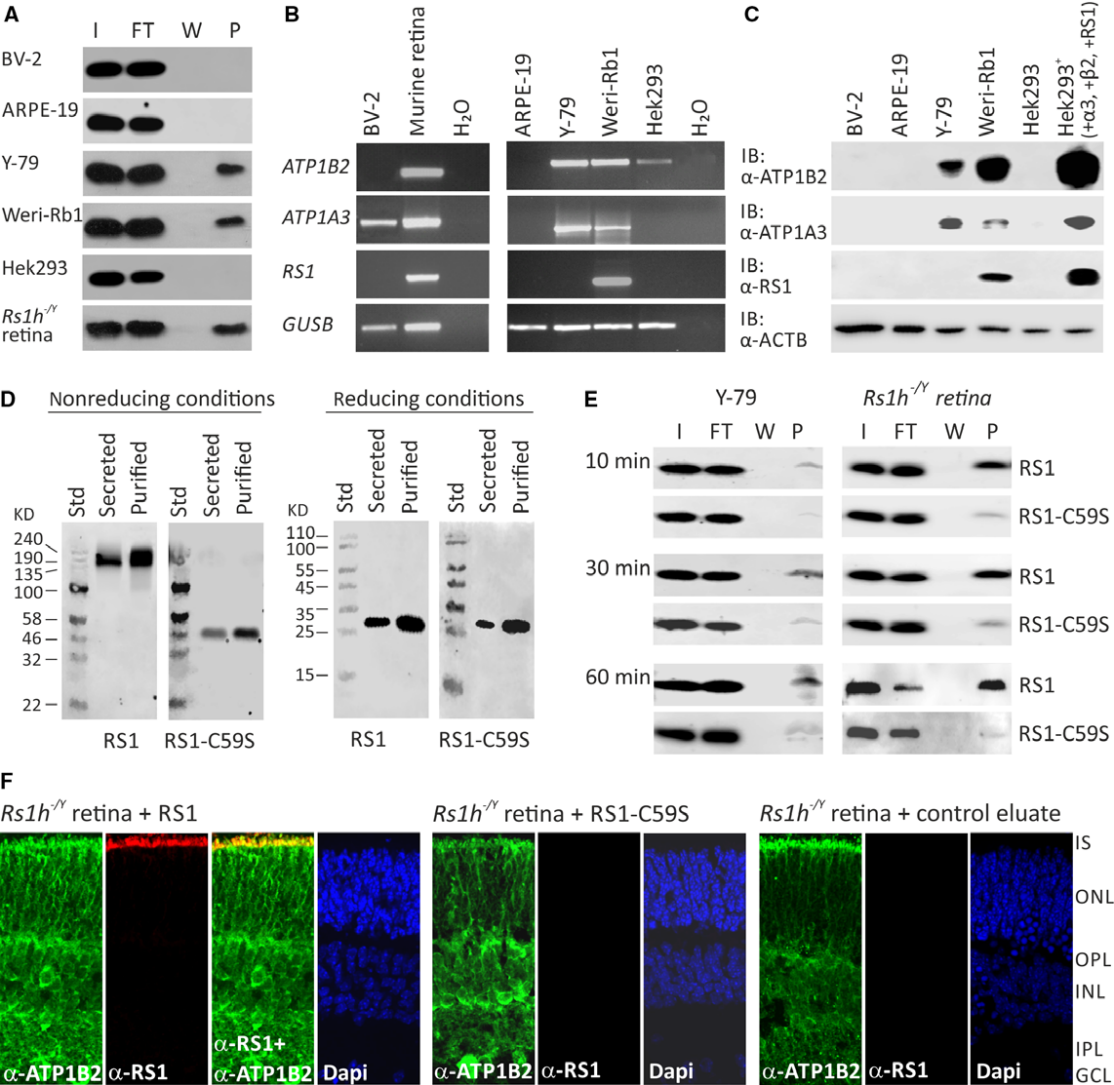
Binding of RS1 variants to retinal cells (**A**) Binding of retinoschisin to cultured retinal cell lines ARPE‐19, Y‐79, Weri‐Rb1 and BV‐2: Cells were incubated for 60 min. with retinoschisin containing supernatant (I, input) of cells stably transfected with a retinoschisin expression vector. Subsequently, cells were centrifuged and supernatant (FT, flowthrough) was discarded. After further washing steps (last supernatant, W), cells were pelleted (pellet, P). Fractions were subjected to Western blot analyses using an antiretinoschisin antibody. Retinoschisin binding to Hek293 cells and murine *Rs1h* ^*−/Y*^ retinal membranes served as negative and positive controls, respectively. (**B**) RT‐PCR analysis of *ATP1B2, ATP1A3* and *RS1* gene expression in cell lines derived from murine microglia (BV‐2), human retinal pigment epithelium (ARPE‐19), human retinoblastoma (Y‐79 and Weri‐Rb1) and human embryonic kidney (Hek293). *GUSB* gene expression was assessed as control for RNA integrity. (**C**) Cell lysates from BV‐2, ARPE‐19, Y‐79, Weri‐Rb1 and Hek293 were subjected to Western blot analyses using antibodies against ATP1B2, ATP1A3 and retinoschisin. Hek293^+^ cells served as positive control. The ACTB immunoblot was performed as loading control. (**D**) Oligomerization of RS1 variants (non‐mutant retinoschisin and RS1‐C59S) before and after purification. About 48 hrs after transfection of Hek293 cells with expression constructs for N‐terminally Myc‐tagged RS1 variants, the cell culture medium (supernatant) was harvested and Myc‐tagged proteins were purified from the supernatant. Aliquots of supernatant and purified RS1 fractions were subjected to SDS‐PAGE under non‐reducing and reducing conditions, followed by Western blot analyses using an antiretinoschisin antibody. (**E**) Binding of RS1 variants to retinal cells. Y‐79 cells and murine *Rs1h* ^*−/Y*^ retinal explants were incubated with purified RS1 variants (I, input) for 10, 30 and 60 min. Cells were centrifuged and supernatant (FT, flowthrough) was discarded. After several washing steps (last supernatant, W), cells were pelleted (pellet, P). Fractions were subjected to Western blot analyses using an antiretinoschisin antibody. (**F**) Localization of recombinant RS1 variants on retinal membranes. *Rs1h* ^*−/Y*^ retinal explants (P10) were incubated for 30 min. with retinoschisin, RS1‐C59S or control protein, the latter purified from supernatant of empty expression vector‐transfected cells. After washing and embedding, cryosections of these explants were subjected to immunohistochemical analyses using antibodies against ATP1B2 and retinoschisin. 4′,6‐Diamidino‐2‐phenylindol (DAPI) staining shows the nuclei of the different retinal layers. IS, inner segments; ONL, outer nuclear layer; OPL, outer plexiform layer; INL, inner nuclear layer; IPL, inner plexiform layer; GCL, ganglion cell layer.

Searching for an *in vitro* model system applicable for analysing the influence of retinoschisin on MAP kinase signalling, we tested different retinal cells including murine microglial cell line BV‐2, human RPE‐derived cell line ARPE‐19, the human retinoblastoma cell lines Y‐79 and Weri‐Rb1 for their capacity to bind retinoschisin (Fig. 2A). In these cells, we also investigated endogenous expression of the retinal Na/K‐ATPase subunits ATP1A3 and ATP1B2, required for anchoring retinoschisin to retinal plasma membranes 23 (Fig. 2B and C).

Retinoschisin binding assays were performed on intact cells as described by 23. Retinoschisin binding to Hek293 cells and crude membranes of *Rs1h* ^*−/Y*^ retinae served as negative and positive controls, respectively 23. Efficient retinoschisin binding was found with Y‐79, Weri‐Rb1 and membranes of *Rs1h* ^*−/Y*^ retinae, but not with ARPE‐19, BV‐2 and Hek293.

Semiquantitative RT‐PCR (Fig. 2B) and Western blot analyses (Fig. 2C) revealed endogenous expression of ATP1A3 and ATP1B2 only in Y‐79 and Weri‐Rb1. Weri‐Rb1 cells also weakly expressed retinoschisin (Fig. 2B and C). In all further analyses, Y‐79 cells were used to assess effects of externally added retinoschisin on intracellular signalling.

### Different binding affinities of normal retinoschisin and the XLRS‐associated mutant protein RS1‐C59S to retinal membranes

To analyse functional properties of retinoschisin, we heterologously expressed RS1 (NM_000330.3) and the XLRS‐associated mutant protein (NM_000330.3(RS1):c.175T>A [p.Cys59Ser], termed RS1‐C59S, in Hek293 cells. The mutation c.175T>A [p.Cys59Ser] is one of the rare XLRS variants which are not subjected to co‐ or post‐translational degradation, but instead are translated and secreted from cells, although not as a stable octamer but instead as a dimer 21, 22. For purification, retinoschisin (normal and mutant) was fused to an N‐terminal Myc tag, which did not influence secretion, oligomerization or binding capacities of the resulting protein (Fig. 2D and E).

Y‐79 cells and *Rs1h* ^*−/Y*^ retinal explants exposed to recombinant retinoschisin for 10, 30 and 60 min. stably bound the externally added retinoschisin, even after only 10 min. of incubation (Fig. 2E). Notably, RS1‐C59S exhibited a strongly reduced binding affinity to Y‐79 cells and *Rs1h* ^*−/Y*^ retinal explants (Fig. 2E).

Immunohistochemical stainings of murine *Rs1h* ^*−/Y*^ retinal explants after treatment with retinoschisin for 30 min. (Fig. 2F) confirmed the binding of externally added recombinant retinoschisin. Recombinant retinoschisin colocalized with the endogenously expressed retinal Na/K‐ATPase of the murine retina at the inner segments of photoreceptor cells. This is in agreement with the localization of retinoschisin in wild‐type retinae 23. In contrast to the known retinoschisin localization, no recombinant retinoschisin was detected in the plexiform layers of the retinal explants, which could possibly be explained by limited diffusion of the externally added retinoschisin through the retinal layers. No retinoschisin staining was observed in immunohistochemical analyses of *Rs1h* ^*−/Y*^ retinal explants treated with RS1‐C59S or control protein (Fig. 2F).

### Extracellular retinoschisin modulates ERK 1/2 signalling in Y‐79 cells and *Rs1h* ^*−/Y*^ murine retinal explants

**Figure 3:**
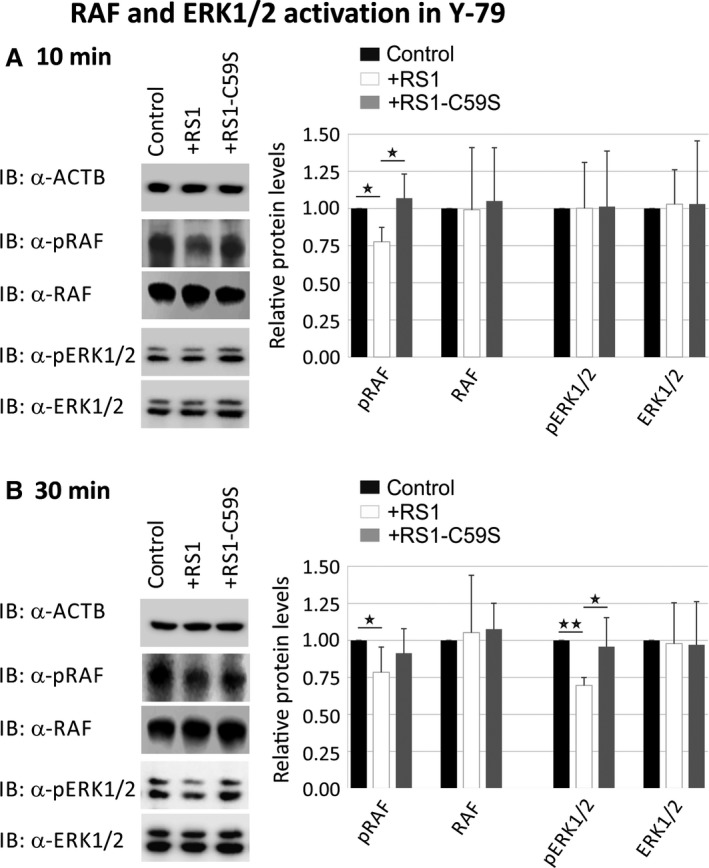
Influence of retinoschisin on the ERK pathway in Y‐79 cells. Retinoschisin‐dependent C‐RAF and ERK1/2 phosphorylation in Y‐79 cells. Y‐79 cells were treated for 10 min. (**A**) or 30 min. (**B**) with retinoschisin, RS1‐C59S or control protein (purified from supernatant of empty expression vector‐transfected cells). Subsequently, the cells were subjected to Western blot analyses with antibodies against phosphorylated C‐RAF (pRAF), total C‐RAF (RAF), phosphorylated ERK1 and ERK2 (pERK1/2), total ERK1 and ERK2 (ERK1/2), as well as ACTB as a control. (**A** and **B**) Densitometric quantification was performed with immunoblots from five independent experiments. Signals for pRAF RAF, pERK1/2 and ERK1/2 were normalized against ACTB and calibrated against the control. Data represent the mean + S.D. Asterisks mark statistically significant (*P < 0.05) and highly significant (**P < 0.01) differences.

**Figure 4:**
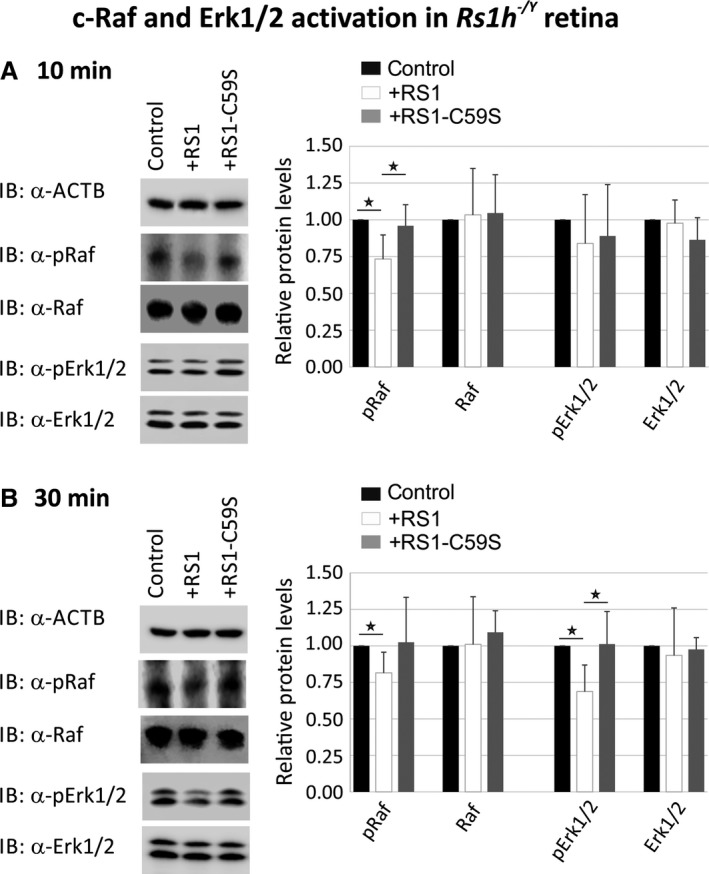
Influence of retinoschisin on the ERK pathway in murine *Rs1h* ^*−/Y*^ retinal explants. Retinoschisin‐dependent c‐Raf and Erk1/2 phosphorylation in murine *Rs1h* ^*−/Y*^ retinal explants. Retinal explants harvested 10 days after birth (P10) were treated for 10 min. (**A**) or 30 min. (**B**) with retinoschisin, RS1‐C59S or control protein (purified from supernatant of empty expression vector‐transfected cells). Subsequently, the retinal lysates were subjected to Western blot analyses with antibodies against phosphorylated c‐Raf (pRaf), total c‐Raf (Raf), phosphorylated Erk1 and Erk2 (pErk1/2), total Erk1 and Erk2 (Erk1/2), as well as ActB as a control. (**A** and **B**) Densitometric quantification was performed with immunoblots from five independent experiments. Signals for pRaf Raf, pErk1/2 and Erk1/2 were normalized against ActB and calibrated against the control. Data represent the mean + S.D. Asterisks mark statistically significant differences (*P < 0.05).

To assess the capacity of retinoschisin to directly modulate intracellular ERK1/2 signalling, we investigated an influence of extracellularly added recombinant retinoschisin (normal and mutant) on phosphorylation of C‐RAF and ERK1/2 in Y‐79 cells (Fig. 3) and *Rs1h* ^*−/Y*^ murine retinal explants (Fig. 4). As control, we applied protein purified from supernatant of mock vector‐transfected cells.

In Y‐79 cells, we observed a down‐regulation of phosphorylated C‐RAF (77.6 ± 9.7%) after 10 min. of treatment with recombinant retinoschisin (Fig. 3A). In contrast, RS1‐C59S failed to inhibit C‐RAF phosphorylation (Fig. 3A). The differences in phosphorylated C‐RAF levels between retinoschisin treatment and control or RS1‐C59S treatment were statistically significant (*P* < 0.05). The effect of retinoschisin on C‐RAF phosphorylation was still evident after 30 min. of treatment with retinoschisin, where C‐RAF phosphorylation was reduced to 78.4 ± 17.0% (Fig. 3B).

In contrast to its effect on C‐RAF in Y‐79 cells, retinoschisin treatment failed to show a significant decrease in ERK1/2 phosphorylation after 10 min. of incubation (Fig. 3A). Thirty minutes of retinoschisin treatment (Fig. 3B), however, reduced ERK activation to around 69.6 ± 5.3% in Y‐79 cells (*P* < 0.05 compared with control protein or RS1‐C59S). No alterations in total C‐RAF and total ERK1/2 protein levels were detected, excluding an effect of retinoschisin on expression or stability of the two proteins (Fig. 3).

MAP kinase signalling in murine *Rs1h* ^*−/Y*^ retinal explants was similarly affected by retinoschisin treatment (Fig. 4). After 10 min. of incubation with retinoschisin, phosphorylated c‐Raf levels were decreased to 73.4 ± 16.3% (Fig. 4A). RS1‐C59S treatment had no effect on c‐Raf phosphorylation. The differences in phosphorylated c‐Raf levels were statistically highly significant (*P* < 0.01) between control and retinoschisin treatment and statistically significant (*P* < 0.05) between retinoschisin and RS1‐C59S treatment. After 30 min. of incubation, the reduction in c‐Raf phosphorylation by retinoschisin treatment was still observable (Fig. 4B): Retinoschisin decreased c‐Raf phosphorylation to 81.4 ± 14.1%, compared with the control.

Erk1/2 phosphorylation was not affected after 10 min. (Fig. 4A), but only after 30 min. of treatment with recombinant retinoschisin (Fig. 4B). In contrast to control or RS1‐C59S treatment, incubation with retinoschisin resulted in a clear reduction in phosphorylated Erk1/2 (68.7 ± 18.2% compared with control, Fig. 4B). Differences in phosphorylated Erk1/2 levels were statistically highly significant (*P* < 0.01) when compared between control and retinoschisin treatment and statistically significant (*P* < 0.05) between retinoschisin and RS1‐C59S treatment. The different treatments caused no changes in total c‐Raf and total Erk1/2 levels in the retinal explants (Fig. 4).

### Extracellular retinoschisin modulates ERK1/2 target gene expression in Y‐79 cells and *Rs1h* ^*−/Y*^ murine retinal explants

**Figure 5:**
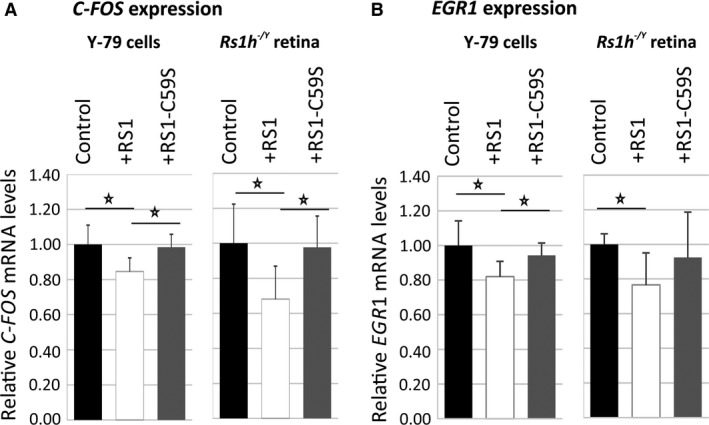
Influence of retinoschisin on the expression of ERK1/2 pathway target genes. Retinoschisin‐dependent *C‐FOS* (**A**) and *EGR1* (**B**) expression in Y‐79 cells and murine *Rs1h* ^*−/Y*^ retinal explants. Cells/retinal explants were treated as described in Figures 3 and 4 for 2 hrs (Y‐79 cells) and 30 min. (murine retinal explants). *C‐FOS* and *EGR1* mRNA expression was determined *via* quantitative real‐time RT‐PCR. Five independent experiments were performed. Results were normalized to *HPRT* transcript levels and calibrated with the control. The mean + S.D. for the five independent experiments is given. Asterisks mark statistically significant differences (*P < 0.05).

Upon treatment with recombinant retinoschisin, a statistically significant down‐regulation of *C‐FOS* mRNA expression was observed in Y‐79 cells (84.6 ± 7.7%) and in *Rs1h* ^*−/Y*^ murine retinal explants (68.1 ± 18.9%) by quantitative RT‐PCR when compared to control treatment (*P* < 0.05, Fig. 5A). In contrast, no prominent decrease in *C‐FOS* transcripts was found after treatment with RS1‐C59S (98.4 ± 7.3% for Y‐79; 97.7 ± 17.9% for retinal explants). The differences between the retinoschisin and the control or RS1‐C59S treatment were statistically significant (*P* < 0.05; Fig. 5A).

Similarly, retinoschisin treatment caused a statistically significant decrease in *EGR1* expression (Fig. 5B): *EGR1* transcript levels were reduced to 82.0 ± 8.8% in Y‐79 cells and to 76.6 ± 18.8% in *Rs1h* ^*−/Y*^ murine retinal explants (*P* < 0.05) when compared to control treatment. No prominent decrease in *EGR1* mRNA levels was found after treatment with RS1‐C59S (94.3 ± 7.1% for Y‐79 cells, *P* < 0.05 compared with retinoschisin treatment, and 92.4 ± 26.2% for retinal explants).

### Retinoschisin is protective for apoptotic events

**Figure 6:**
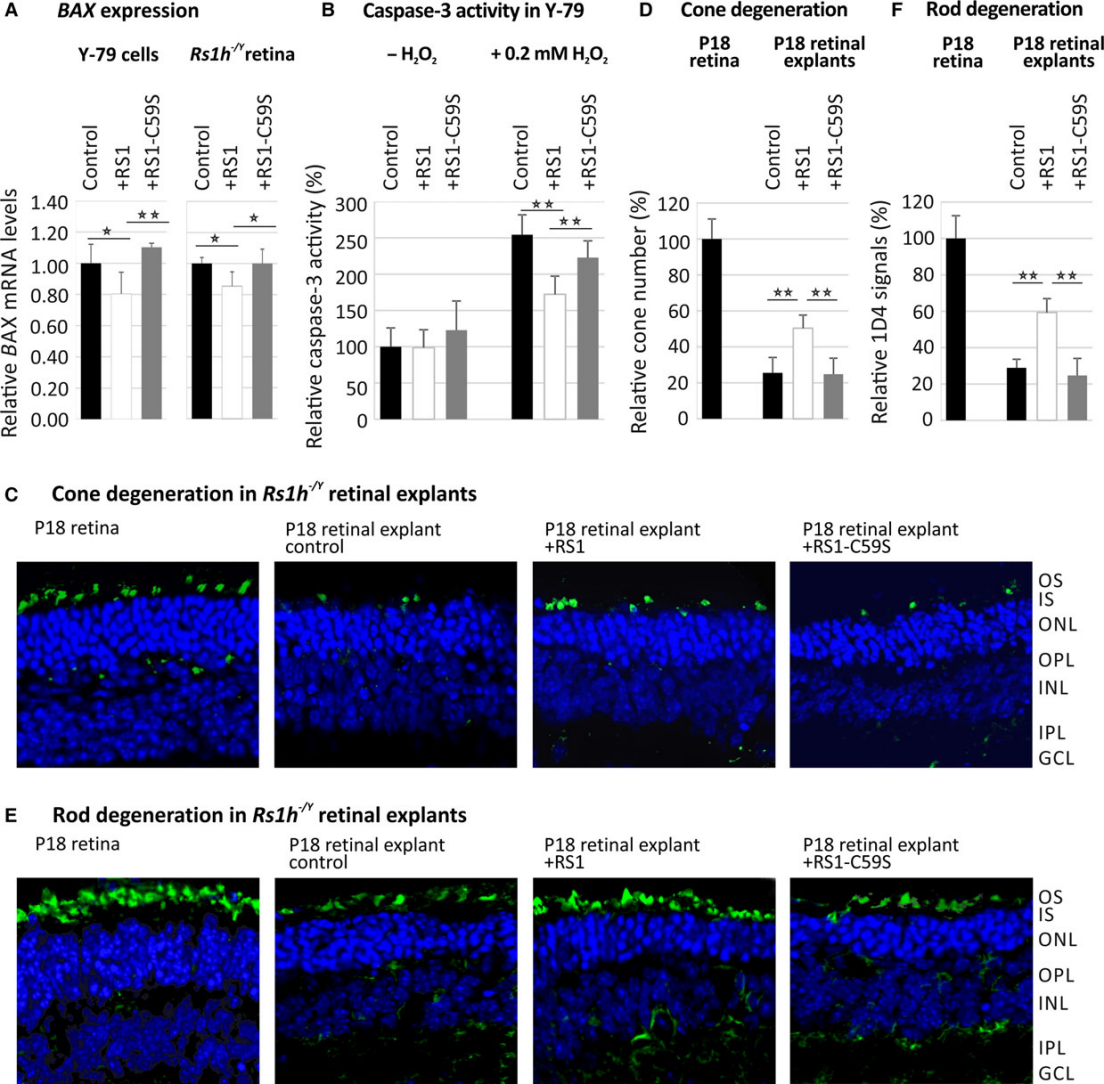
Influence of retinoschisin on apoptosis. (**A**) Retinoschisin‐dependent *BAX* expression in Y‐79 cells and murine *Rs1h* ^*−/Y*^ retinal explants. Y‐79 cells or retinal explants were treated with retinoschisin, RS1‐C59S or control protein for 20 hrs or 30 min., respectively. *BAX* mRNA expression was determined *via* quantitative real‐time RT‐PCR. Five independent experiments were performed. Results were normalized to *HPRT* transcript levels and calibrated with the control. The mean + S.D. for the five independent experiments is given. Asterisks mark statistically significant (*P < 0.05) and highly significant (**P < 0.01) differences. (**B**) Retinoschisin‐dependent activation of caspase‐3 in Y‐79 cells subjected to oxidative stress. Y‐79 cells, exposed to retinoschisin, RS1‐C59S or control protein were treated with 0.2 mM H_2_O_2_ for 2 hrs. About 18 hrs later, apoptosis was assayed by following caspase‐3‐specific proteolytic activity. Data represent the mean + S.D. of six independent experiments. Asterisks mark statistically highly significant differences (**P < 0.01). (**C**–**F**) Retinoschisin‐dependent photoreceptor degeneration in murine *Rs1h* ^*−/Y*^ retinal explants. Retinal explants harvested 18 days after birth (P18) were cultured for 1 week in medium containing retinoschisin, RS1‐C59S or control protein (purified from supernatant of empty expression vector‐transfected cells). After washing and embedding, cryosections of these explants were subjected to staining for nuclei, cones and rods. OS, outer segments; IS, inner segments; ONL, outer nuclear layer; OPL, outer plexiform layer; INL, inner nuclear layer; IPL, inner plexiform layer; GCL, ganglion cell layer. DAPI staining shows the nuclei of the different retinal layers. **(C)** Alexa488‐conjugated peanut agglutinin (PNA) staining was applied to visualize cones. **(D)** The total number of cones per analyzed section was counted after staining with PNA. **(E)** Anti‐Rho‐1D4 antibody staining was applied to visualize rod specific Rhodopsin. **(F)** Rhodopsin signals per analyzed section were measured using ImageJ (imagej.nih.gov). Data represent the mean + SD. Asterisks mark statistically highly significant differences (**P < 0.01).

Mitogen‐activated protein kinase signalling is an important mediator and regulator of several physiological processes implicated in XLRS pathogenesis 41, 43, 44, 45, 46, 47, 48, 49, 50, 62. As one characteristic feature of *Rs1h* ^*−/Y*^ mice is an early photoreceptor degeneration due to apoptotic cell death 63, we analysed the influence of retinoschisin on the expression of apoptosis marker BAX in Y‐79 cells and murine *Rs1h* ^*−/Y*^ retinal explants. Additionally, we assessed retinoschisin‐dependent caspase‐3 activation as a prominent marker for apoptosis induction 64, 65, 66 in Y‐79 cells, as well as the influence of recombinant retinoschisin on cone degeneration in murine *Rs1h* ^*−/Y*^ retinal explants (Fig. 6).

Short time exposure to retinoschisin caused no change in *BAX* transcript levels in Y‐79 cells (data not shown). After 20 hrs of incubation, however, *BAX* transcript levels were significantly decreased (77.6 ± 14.0%) compared with control (*P* < 0.05) or RS1‐C59S treatment (*P* < 0.01, Fig. 6A). In *Rs1h* ^*−/Y*^ retinal explants, 30 min. of incubation with retinoschisin reduced *BAX* transcript levels to 85.4 ± 9.3% (*P* < 0.01 compared with control and RS1‐C59S‐treated retinae, Fig. 6A). In contrast, incubation with RS1‐C59S had no significant effect on *BAX* expression in Y‐79 cells (109.5 ± 2.6%) or retinal explants (103.3 ± 11.8%, Fig. 6A).

Caspases were shown to play a prominent role in photoreceptor cell death in the retinoschisin‐deficient mouse 63. We thus followed caspase‐3 activation in Y‐79 cells upon stress‐induced apoptosis after treatment with 0.2 mM hydrogen peroxide [H_2_O_2_] 64, 65, 66. H_2_O_2_ treatment caused an about 2.5‐fold increase in caspase‐3 activity compared with unstimulated cells (Fig. 6B). Notably, in cells stimulated with H_2_O_2_, retinoschisin strongly decreased caspase‐3 activation (67.6 ± 9.9%) compared with control protein or RS1‐C59S (*P* < 0.01). RS1‐C59S led to a slight, albeit statistically not significant, reduction in caspase‐3 activity (87.6 ± 9.1%, *P* = 0.59) in H_2_O_2_‐treated cells.

Finally, we addressed retinoschisin‐dependent photoreceptor survival by following cone and rod degeneration in murine *Rs1h* ^*−/Y*^ retinal explants 63. Photoreceptor cell death in *Rs1h* ^*−/Y*^ mice is triggered by apoptotic events initiated around 14 days after birth (P14, 63). We isolated *Rs1h* ^*−/Y*^ retinae 18 days after birth (P18) and incubated them in medium containing retinoschisin, RS1‐C59S or control protein. One week of cultivation resulted in a strong degeneration of retinal explants, shown by a markedly decreased thickness of the central retina and a significant reduction in photoreceptor cells (Fig. 6C–F). More specifically, compared with untreated retinae, 1 week of cultivation reduced the number of cones to around 25% in control and RS1‐C59S‐treated explants (25.6 ± 8.5% for control and 24.8 ± 8.9% for RS1‐C59S treatment (Fig. 6C and D). Notably, in explants treated with retinoschisin, the cone number was decreased to only about 50% (50.3 ± 7.3% compared with untreated retinae), with a statistically highly significant difference to control and RS1‐C59S‐treated explants (*P* < 0.01). Investigations on rod degeneration revealed similar results (Fig. 6E and F). After 1 week of cultivation, rod signals were reduced to around 24.8 ± 9.3% for control treated and to 28.9 ± 4.7% for RS1‐C59S‐treated explants. Treatment with retinoschisin lead to a rod signal decrease of only 59.3 ± 7.6% compared with untreated retinae, with statistically highly significant differences to control and RS1‐C59S‐treated explants (*P* < 0.01).

## Discussion

In this study, we investigated the role of retinoschisin in the regulation of intracellular MAP kinase signalling. Firstly, our experiments confirmed strongly increased MAP kinase signalling in early retinal development of *Rs1h* ^*−/Y*^ mice. Secondly, we demonstrated that retinoschisin binding directly decreased phosphorylation of C‐RAF and MAP kinases ERK1 and ERK2, as well as expression of the MAP kinase target genes *C‐FOS* and *EGR1* in a retinal (Y‐79) cell line and in murine *Rs1h* ^*−/Y*^ retinal explants. Thirdly, our data suggest a protective effect of retinoschisin against apoptotic cell death in Y‐79 cells and *Rs1h* ^*−/Y*^ retinal explants. As a stringent control, the XLRS mutant RS1‐C59S was deficient in binding to retinal membranes, and failed to reveal regulation on MAP kinase signalling or effects on apoptosis. Together, our results demonstrate that retinoschisin is a novel regulator of intracellular signalling and protects retinal cells from apoptosis. We suggest that aberrant MAP kinase signalling due to retinoschisin deficiency could be an initial trigger in XLRS pathogenesis.

In recent years, the importance of MAP kinase signalling in retinal development and homeostasis has attracted increasing attention 67, 68, 69. Not surprisingly, several retinal dystrophies such as age‐related macular degeneration 70, 71, 72, diabetic retinopathy 73 or retinitis pigmentosa 74, 75 were linked to malfunctioning MAP kinase pathways. Aberrant MAP kinase signalling was also observed during early retinal development in the XLRS mouse model 40. Our study verified the earlier observations from Gehrig *et al*. 40 by showing increased activation of central constituents of the ERK pathway, c‐Raf and Erk1/2 57, in *Rs1h* ^*−/Y*^ retinae of 7‐, 10‐ and 14‐day‐old mice. Furthermore, we showed up‐regulation of prominent target genes of MAP kinase signalling, namely *c‐Fos* and *Egr1* 56, indicating an early and sustained alteration in MAP kinase signalling in disease development of *Rs1h* ^*−/Y*^ mice.

To assess whether retinoschisin has the capacity to directly modulate MAP kinase signalling, we investigated the effect of recombinant retinoschisin on activation of the ERK pathway in two retinal model systems; the human retinoblastoma cell line Y‐79 and *Rs1h* ^*−/Y*^ murine retinal explants, both capable to bind extracellularly added retinoschisin due to an endogenous expression of the NA/K‐ATPase subunits α3 and β2. The addition of recombinant retinoschisin had an immediate and significant influence on MAP kinase signalling in these two model systems, reflected by decreased C‐RAF and ERK1/2 phosphorylation. C‐RAF activation (10 min.) occurred before ERK1/2 phosphorylation (30 min. after addition of retinoschisin), in agreement with the established sequence of C‐RAF and ERK1/2 activation in the ERK signalling cascade 57, 76. Subsequently, retinoschisin treatment also induced down‐regulation of *C‐FOS* and *EGR1* expression in Y79 cells and *Rs1h* ^*−/Y*^ murine retinal explants. These results establish retinoschisin as an important regulator of the MAP kinase pathway in retinal cells.

The contribution of MAP kinase signalling to various disease processes can be explained by its key role in the regulation of complex physiological processes such as apoptosis, adhesion, proliferation, differentiation or development 41, 44, 49. For instance, several studies showed a pro‐apoptotic effect of ERK activation specifically connected to neuronal cells, for example in neurodegenerative disease processes 77, 78, 79, 80. Of note, a characteristic increase in ERK1/2 activation with an effect size similar to our results has been described for early disease stages of Alzheimer's disease with 25% less ERK1/2 activation in temporal cortex of healthy individuals compared with patients 81, or of ocular ischaemic syndrome where 29% less ERK1 and 21% less ERK2 activation in murine retinae of control mice were found when compared to a mouse model of ocular ischaemic syndrome 82. Additionally, comparably small alterations in MAP kinase signalling, related to cellular survival, were found in natural killer cells of chronic fatigue syndrome 83 and in lymphocytes of patients with Alzheimer's and Parkinson's disease 84.

Consistently, we demonstrate a protective influence of retinoschisin against apoptosis: Transcript levels of the pro‐apoptotic BAX protein 85, 86 were down‐regulated in Y‐79 cells and *Rs1h* ^*−/Y*^ retinae exposed to recombinant retinoschisin. Furthermore, in Y‐79 cells subjected to oxidative stress, caspase‐3 activity, a marker for the induction of apoptosis 65, was significantly decreased by retinoschisin. Similarly, apoptosis‐induced cone and rod degeneration 63 in murine *Rs1h* ^*−/Y*^ retinal explants was strongly reduced in the presence of recombinant retinoschisin. Further studies are required to verify the direct contribution of increased MAP kinase activation to photoreceptor apoptosis in XLRS pathogenesis. Nevertheless, considering the current state of knowledge on the pathological role of MAP kinase activation in neurodegeneration 77, 78, 79, 80, 81, 82, we speculate that increased MAP kinase signalling due to retinoschisin deficiency can induce or contribute to XLRS‐associated neurodegenerative processes in humans, and apoptotic photoreceptor degeneration in the XLRS mouse model 40.

Our investigations included studies on the functionality of the XLRS‐associated retinoschisin mutant, RS1‐C59S. Unlike most RS1 mutants, RS1‐C59S is translated and secreted from cells, but with defective oligomerization 21, 22. The functional consequences of this structural alteration have not been elucidated, so far. Here, we show that RS1‐C59S cannot bind to retinal membranes and can thus not fulfil its function as a regulator of intracellular signalling.

The present data do not allow elucidation of how extracellular retinoschisin binding affects intracellular MAP kinase signalling. Previous analysis identified the retinal Na/K‐ATPase as the specific binding partner for retinoschisin on retinal membranes 14, 23. Several groups reported that in addition to their function as an ion pump 87, 88, Na/K‐ATPases are important regulators of intracellular MAP kinase signalling 31, 89, 90, 91, although the exact mechanism of signal transduction from Na/K‐ATPases to the MAP cascade is under debate 30, 92, 93, 94, 95, 96. It would thus be conceivable that retinoschisin modulates the capacity of the Na/K‐ATPase to regulate intracellular signalling. A disruption of this retinoschisin‐Na/K‐ATPase signalosome complex by retinoschisin deficiency could therefore result in defective MAP kinase regulation by the Na/K‐ATPase.

Taken together, we provide evidence that retinoschisin is a novel regulator of MAP kinase signalling in the retina with the capacity to protect cells against apoptotic cell death. We suggest that disturbances of intracellular MAP kinase signalling by retinoschisin deficiency might be one of the initial steps in XLRS pathology. Thus, our data could provide a novel basis for considerations to therapeutic treatments for this progressive and currently untreatable disease.

## Conflict of interest

None declared.

## Supporting information


**Figure S1** Purity of Myc‐tagged RS1 proteins.

**Table S1** Primers used in RNA analyses and expression cloning.
