# Supplementary figures and images for: The X‐linked juvenile retinoschisis protein retinoschisin is a novel regulator of mitogen‐activated protein kinase signalling and apoptosis in the retina
Source: J Cell Mol Med. 2016 Dec 20;21(4):768–80. doi: 10.1111/jcmm.13019 (PMC5345684; doi:10.1111/jcmm.13019)

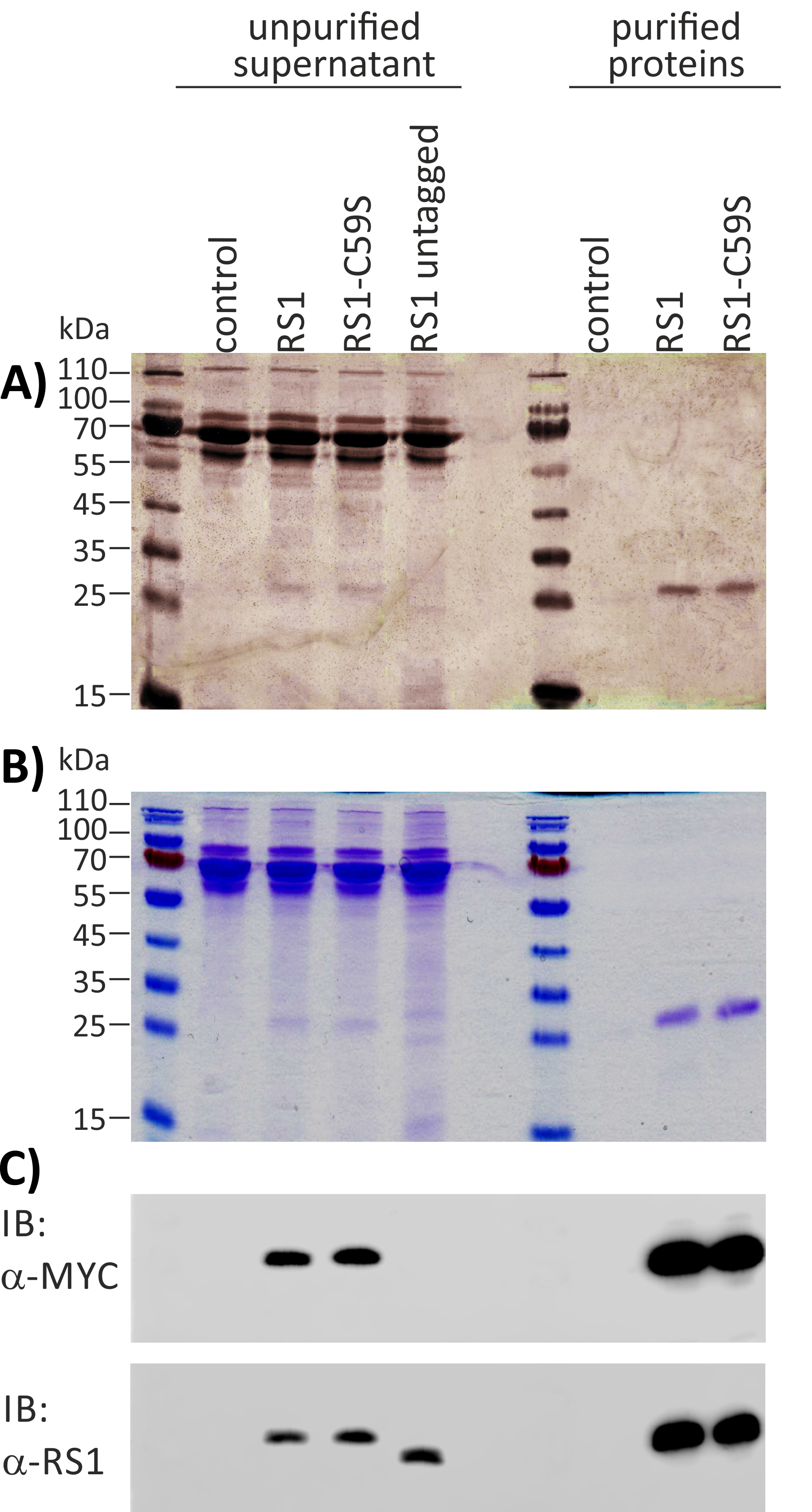

Supplement: Supplementary file 1 — Figure S1 Purity of Myc‐tagged RS1 proteins. [file JCMM-21-768-s001.tif]
