# Supplementary material for: The X‐linked juvenile retinoschisis protein retinoschisin is a novel regulator of mitogen‐activated protein kinase signalling and apoptosis in the retina
Source: J Cell Mol Med. 2016 Dec 20;21(4):768–80. doi: 10.1111/jcmm.13019 (PMC5345684; doi:10.1111/jcmm.13019)
Supplement: Supplementary file 2 — Table S1 Primers used in RNA analyses and expression cloning. [file JCMM-21-768-s002.pdf]

Primer Name	Sequence (5' to 3')	Use
hATP1A3-Ex1-F	AGA TGG GGG ACA AGA AAG ATG AC	semiquantitative PCR against human target genes
hATP1A3-Ex9-R	CCT CAA GCC AGG TGA TCC G	
hATP1B2-Ex-4-F	CAA ACG TGC CTG CCA ATT CA	
hATP1B2-Ex7-R	TCC ACA GGA GAG AGA TGG GG	
hRS1-ex4-5-F	AGT CAA GGC TTT GGG TGT GCC TG	
hRS1-ex6-R	TGG CAA TGC GGA CGT GCC AG	
GusB-Ex11-12_F	GGT TGG AGA GCT CAT TTG GA	
GusB-Ex11-12_	CAC GCA GGT GGT ATC AGT CTT	
mATP1A3-Ex1-F	CGC TCA TCA GTC TGA ACG CC	semiquantitative PCR against murine target genes
mATP1A3-Ex6-R	CGG TCA CCA CCC TTG ATC TC	
mATP1B2-Ex-1-F	CCC TTT CTA TCG CAG TCG G	
mATP1B2-Ex4-R	AAC ATG CTG ACC CCA GCT TT	
RT_mRs1_F2	GTC AAG GTT TTG GGT GTG CT	
RT_mRs1_R2	AAC TCC GGTCTG AGT TTC CA	
RT_mGusb_F1	CCC TAA AAT GGA GTG CGT GT	
RT_mGusb_R1	AAT GGG CAC TGT TGA TCC TC	
hFOS-qRT-F	ACT ACC ACT CAC CCG CAG AC	quantitative real-time RT PCR against human target genes
hFOS-qRT-R	CCA GGT CCG TGC AGA AGT	
hEGR-qRT-F	AGC CCT ACG AGC ACC TGA C	
hEGR1-qRT-R	GGT TTG GCT GGG GTA ACT G	
hMYC-qRT-F	GCT GCT TAG ACG CTG GAT TT	
hMYC-qRT-R	TAA CGT TGA GGG GCA TCG	
hBAX-qRT-F	ATG TTT TCT GAC GGC AAC TTC	
hBAX-qRT-R	ATC AGT TCC GGC ACC TTG	
hHPRT-qRT-F	TGA CCT TGA TTT ATT TTG CAT ACC	
hHPRT-qRT-R	CGA GCA AGA CGT TCA GTC CT	
mFOS-qRT-F	GGG ACA GCC TTT CCT ACT ACC	quantitative real-time RT PCR against murine target genes
mFOS-qRT-R	AGA TCT GCG CAA AAG TCC TG	
mEGR1-qRT-F	CCT ATG AGC ACC TGA CCA CA	
mEGR1-qRT-R	TCG TTT GGC TGG GAT AAC TC	
mMYC-qRT-F	CCT AGT GCT GCA TGA GGA GA	
mMYC-qRT-R	TCC ACA GAC ACC ACA TCA ATT	
mBAX-qRT-F	GTG AGC GGC TGC TTG TCT	
mBAX-qRT-R	GGT CCC GAA GTA GGA GAG GA	
mHPRT-qRT-F	TGA CAC TGG TAA AAC AAT GCA	
mHPRT-qRT-R	TCC TTT TCA CCA GCA AGC TTG	
RS1-EcoRI-F	GAA TTC ATG TCA CGC AAG ATA GAA GGC TTT TTG	Expression cloning *RS1*
RS1-XhoI-R	CTC GAG TCA GGC ACA CTT GCT GAC GCA CTC	
RS1-C59S-mut-F	CCA CCT CCT TGG AC**A** GTA TAC CAG AAT GCC	site directed mutagenesis
RS1-C59S-mut-R	GGC ATT CTG GTA TAC **T**GT CCA AGG AGG TGG	
RS1-EcoRI-F	GAA TTC ATG TCA CGC AAG ATA GAA GGC TTT TTG	cloning of N-termial part of Myc-tagged *RS1* variants
RS1-n-termMyc-R	***TGA TCA*** **ATT TCT GCT C**CG ATA ATC CCA ATG TGG CTT CAT AGC CAA AGA GA	
RS1-ntermMyc-F	***TGA TCA*** **GTG AGG AAG ATC TG**T CTA CCG AGG ATG AAG GCG AGG ACC CCT	cloning of C-termial part of Myc-tagged *RS1* variants
RS1-XhoI-R	CTC GAG TCA GGC ACA CTT GCT GAC GCA CTC	

Supplementary Table 1 Primers used in RNA analyses and expression cloning.
